# Exploring Generative Artificial Intelligence-Assisted Medical Education: Assessing Case-Based Learning for Medical Students

**DOI:** 10.7759/cureus.51961

**Published:** 2024-01-09

**Authors:** Matthew Sauder, Tara Tritsch, Vijay Rajput, Gary Schwartz, Mohammadali M Shoja

**Affiliations:** 1 Medical Education, Dr. Kiran C. Patel College of Allopathic Medicine, Nova Southeastern University, Fort Lauderdale, USA

**Keywords:** problem-based learning, medical education, generative artificial intelligence, chat generative pre-trained transformer, case-based learning

## Abstract

The recent public release of generative artificial intelligence (GenAI) has brought fresh excitement by making access to GenAI for medical education easier than ever before. It is now incumbent upon both students and faculty to determine the optimal role of GenAI within the medical school curriculum. Given the promise and limitations of GenAI, this study aims to assess the current capabilities of a GenAI (Chat Generative Pre-trained Transformer, ChatGPT), specifically within the framework of a pre-clerkship case-based active learning curriculum. The role of GenAI is explored by evaluating its performance in generating educational materials, creating medical assessment questions, answering medical queries, and engaging in clinical reasoning by prompting it to respond to a problem-based learning scenario. Our results demonstrated that GenAI addressed epidemiology, diagnosis, and treatment questions well. However, there were still instances where it failed to provide comprehensive answers. Responses from GenAI might offer essential information, hint at the need for further inquiry, or sometimes omit critical details. GenAI struggled with generating information on complex topics, raising a significant concern when using it as a 'search engine' for medical student queries. This creates uncertainty for students regarding potentially missed critical information. With the increasing integration of GenAI into medical education, it is imperative for faculty to become well-versed in both its advantages and limitations. This awareness will enable them to educate students on using GenAI effectively in medical education.

## Introduction

Since the inception of artificial intelligence (AI), there has been interest in leveraging its potential within the field of medicine. Implementing the promise of AI has been pursued in nearly all areas of healthcare, with notable progress in many specialties, such as radiology and ophthalmology [1,2]. The field of medical education has similarly been interested in harnessing the power of AI to improve student learning and streamline administrative responsibilities [3-5]. The recent public release of generative AI (GenAI), Chat Generative Pre-trained Transformer (ChatGPT), has brought fresh excitement to this arena, making access to GenAI for medical education easier than ever. GenAI falls broadly in the category of machine learning and large language models but differs from other AI counterparts in that it produces new content in text, audio, images, simulations, and videos [4,6]. With these capabilities, it is understandable that there is both excitement and hesitation regarding applying GenAI in medical education.

Focusing specifically on education within medical school, there are numerous ways in which GenAI can reshape current curriculum delivery and learning models and norms. While the rate of progress will not be homogenous across the following areas, GenAI is certain to impact educator content creation, student personalized learning, information gathering, and learner assessment [4,7]. For educator content creation, GenAI can help produce educational materials, exercises, and clinical cases based on provided prompts and information [8]. Students can utilize GenAI, albeit not originally designed for this purpose, as a sort of "search engine" to obtain answers to their specific questions with the additional benefit of analyzing their performance and adapting to their individual needs. Furthermore, GenAI can be used to develop and grade quizzes and other assessments, allowing educators to spend more time on other pursuits.

While the potential benefits of GenAI are expansive, several limitations have arisen. One major area of concern is intellectual property rights [9]. The legal system is in the process of sorting out how the outcomes of GenAI fit within existing copyright law [10]. Additionally, school policies regarding plagiarism will be adapted due to the prevalence of GenAI [5]. Another noted limitation of GenAI is the bias of the algorithms and the bias of the information that feeds into the algorithm [11,12]. Ultimately, GenAI is currently still in its early phases and has documented instances of limited, misleading, and incorrect information. Hallucinations, for instance, are events in which GenAI extrapolates conclusions without sufficient supporting data [13-15]. Due to these limitations, many have advocated caution regarding using GenAI, and numerous institutions have made recommendations regarding its appropriate use. Given the promise and limitations of GenAI, this paper seeks to assess its current capabilities in the framework of a pre-clerkship case-based learning curriculum.

## Materials and methods

A systematic approach was utilized to assess the capabilities of ChatGPT-4 in generating educational materials, creating medical assessment questions, answering medical questions, and reasoning through case-based learning scenarios. For educational materials, ChatGPT-4 was prompted to generate study guides for the medical conditions of each organ system (integumentary, skeletal, muscular, nervous, endocrine, cardiovascular, lymphatic, respiratory, digestive, urinary, and reproductive systems). For creating medical assessment questions, ChatGPT-4 was prompted to generate National Board of Medical Examiners (NBME)-)-style questions about the same medical conditions as for the summaries. For answering medical questions, specific questions were generated to assess Chat-GPT-4's ability to answer epidemiological, diagnostic, and treatment questions. The conditions selected and the precise language utilized are summarized in Tables 1-2.

**Table 1:** Selected representative diseases by organ system

Organ System	Selected Disease
Integumentary	Melanoma
Skeletal	Osteosarcoma
Muscular	Duchenne’s Muscular Dystrophy
Nervous	Multiple Sclerosis
Endocrine	Type 2 Diabetes Mellitus
Cardiovascular	Congestive Heart Failure
Lymphatic	Acute Lymphoblastic Leukemia
Respiratory	Asthma
Digestive	Crohn’s Disease
Urinary	Benign Prostate Hyperplasia (BPH)
Reproductive	Endometriosis

**Table 2:** Prompt templates for Chat Generative Pre-trained Transformer (ChatGPT)

Prompts for study material and test question generation	Prompts for epidemiological, diagnostic, and treatment questions	
Prompt for ChatGPT to generate study material: Please create a comprehensive study document for medical students learning about (selected disease) for board exams.	What are the relevant epidemiological data for (selected disease)?	
	What are the diagnostic criteria for (selected disease)?	
Prompt for ChatGPT to generate test questions: Please generate three board exam-relevant test questions about (selected disease) appropriate for medical students.		
	What are the treatment options for (selected disease)?	

ChatGPT was fed a representative case-based learning scenario from Edmunds et al. [16] and asked to address questions about the prompts to assess clinical reasoning within a case-based learning environment. A qualitative analysis of the performance of ChatGPT-4 with representative dialogue was performed.

## Results

Evaluation of GenAI in a medical school preclinic curriculum

ChatGPT generated responses for all the prompts and selected diseases for each organ system. Regarding generating a study document, ChatGPT generated responses that typically involved seven sections: introduction, epidemiology, pathophysiology, clinical presentation, diagnosis, treatment, and conclusion. For generating test questions, ChatGPT was also able to generate questions for each selected disease. The test questions were formatted as multiple-choice questions with four possible answers labeled "a-d." In addition, the correct answer was listed below the question-and-answer choices. When prompted to answer a question on the epidemiology of the 11 selected diseases, ChatGPT was able to generate a response for each prompt. Although responses varied in their organization, they were generally divided into subsections about the following categories: introduction, prevalence, age and gender, age of onset, ethnicity, genetics, geography, risk factors, prognostic factors, comorbidities, mortality, complications, and conclusion.

**Figure 1:**
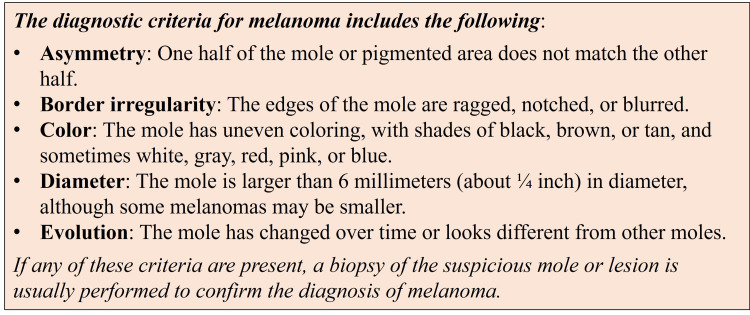
Chat Generative Pre-trained Transformer (ChatGPT) providing diagnostic criteria for melanoma

ChatGPT was able to generate a response for all questions asking about the diagnostic criteria for the 11 selected diseases. Responses did not follow a standard format but were tailored to the diagnostic criteria for each selected disease. A representative example of a response to the diagnostic criteria of melanoma is shown in Figure 1.

**Figure 2:**
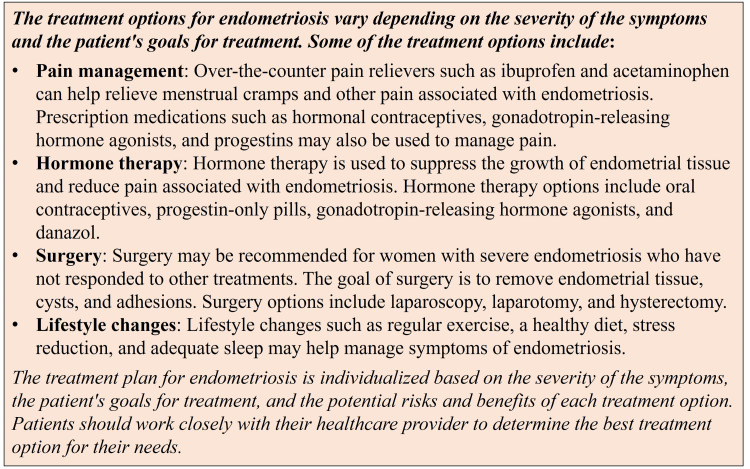
Chat Generative Pre-trained Transformer (ChatGPT) providing treatment options for endometriosis

ChatGPT was also able to generate a response to the question asking about treatment for each of the 11 selected diseases. Similar to the responses for the diagnostic criteria prompt, responses for treatment were variable and tailored to the specific selected disease. A representative example of a response for treating endometriosis is shown in Figure 2.

Evaluation of GenAI for problem-based learning in the preclinic curriculum

**Figure 3:**
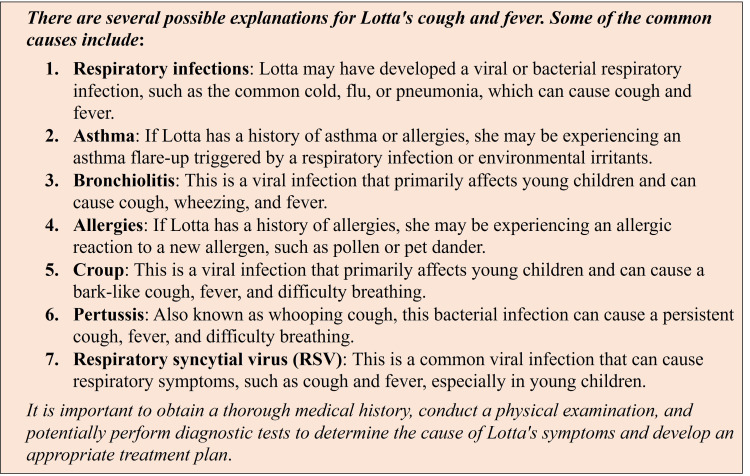
Chat Generative Pre-trained Transformer (ChatGPT) providing differential diagnoses for cough and fever

ChatGPT was able to generate a response to each question throughout the problem-based learning scenario. Additionally, it was able to generate responses that reflect the information that accumulates via the sequential reveal of the case. Prompts, questions, and responses generated for the problem-based learning scenario questions can be found in the Appendix (Tables 3, 4). One example of a response to a question prompting discussion of a differential diagnosis based on initial symptoms is shown in Figure 3.

In addition to providing answers regarding differential diagnosis, ChatGPT was able to answer questions regarding gathering a patient's history, determining what to assess for on physical examination, selecting appropriate next steps in diagnosis and treatment, and describing the basic science behind disease and treatment. One limitation of ChatGPT in responding to this problem-based learning scenario is its inability to intake radiological images. This became of note when the scenario included a chest x-ray and a photograph of the culture results. Despite this limitation, the scenario included the chest X-ray, and culture descriptors that enabled ChatGPT to interpret the radiology and lab findings indirectly.

## Discussion

Although still in its infancy, ChatGPT and other GenAIs are powerful tools with the potential to reshape medical education. Khan et al. outline seven roles for GenAI in medical education: automated scoring, teaching assistance, personalized learning, research assistance, quick access to information, generating case scenarios, and creating content to facilitate learning [17]. This provides an opportunity for both educators and students to streamline the educational process and gain back valuable time that can be spent on other academic, clinical, and research pursuits. Despite these advantages, the authors warn that GenAI, specifically referencing ChatGPT, is not without fault [17]. They note that ChatGPT can ignore context, leading to extraneous detail in the response. Additionally, it was noted that ChatGPT lacks more recent data, which is a concern given the rapid expansion of medical knowledge and clinically relevant changes that have occurred since [17]. The present study looked at the current ability of ChatGPT to act as a generator of study materials from which medical students can study, a "search engine" to answer questions from medical students, a generator of test questions for educators, and as an interactive tool to complement learning in case-based learning scenarios.

Our study demonstrated that GenAI is currently not comprehensive enough to generate information to serve as a complete study guide for medical students. On the other hand, it did show tremendous promise in answering prompts regarding epidemiological, diagnostic, and treatment questions. GenAI also showed potential in developing test questions appropriate for medical student learning and preparation for board exams. Lastly, GenAI showed promise as an interactive tool to guide students through case-based learning scenarios. Given the findings of this study and the fact that GenAI is a new technology with the potential for significant improvement moving forward, students and faculty should explore ways to integrate GenAI into the learning process.

Additionally, faculty should become well-versed in the advantages and shortcomings of GenAI to appropriately educate students on how it should be optimally used in medical education. A guideline set forth by the University of Southern California advises faculty members to educate themselves about GenAI proactively and to provide students with information about the technology's strengths and limitations [18]. In his commentary, Lee delves into both the potential benefits and shortcomings of ChatGPT in medical education [19]. He notes that GenAI can lead to improved efficiency, student engagement, and performance outcomes by providing opportunities for interactive simulations that educate and engage simultaneously. On the other hand, Lee notes concern over ethical concerns regarding mistakes produced by GenAI [19].

Generation of study materials

Regarding being a generator of study materials, although ChatGPT provides accurate and relevant information, it is not as comprehensive as existing information stores commonly studied by medical students. Current and commonly used study resources offer more information for medical students to study. For instance, the study guide generated by ChatGPT for melanoma did not reference its association with the S-100 tumor marker, various forms of melanoma (superficial spreading, nodular, lentigo maligna, and acral lentiginous), and that patients with v-Raf murine sarcoma viral oncogene homolog B (BRAF) V600E mutations can stand to benefit from treatment with a BRAF kinase inhibitor. As such, students utilizing ChatGPT as a primary study resource may not be exposed to information critical to understanding the disease, disease diagnosis, and disease management. Furthermore, students would not be exposed to commonly tested information on board exams.

Given the current limitations of GenAI in creating study guides, it is advisable not to rely on GenAI as the primary study resource. This is especially true when quality existing resources are available and contain more thorough and board-relevant information. In a study, Johnson et al. prompted ChatGPT to answer 180 questions submitted by 33 physicians representing 17 different specialties [20]. Among the answers, 53.5% were comprehensive, indicating that ChatGPT addressed all aspects of the questions and provided additional context beyond expectations [20]. However, just under half of the answers omitted significant portions or provided only the minimum required information to be considered complete. For a student seeking to grasp the nuances of clinical medicine, this level of missing content could present a reliability challenge and would discourage them from solely relying on GenAI responses.

Clinical reasoning and answering medical queries

Although ChatGPT did not generate comprehensive study guides for medical school, the GenAI addressed specific questions regarding epidemiology, diagnosis, and treatment better. For instance, taking the responses to multiple sclerosis as an example, GenAI correctly identified its association with age and gender, its complex genetics, and its significant impact on quality of life. Similarly, it outlined both appropriate diagnostic criteria and treatment options. Despite addressing these aspects, some key points were still not addressed. For instance, the GenAI did not list the exact treatment when referencing disease-modifying agents in managing multiple sclerosis. This leads to a key issue with using GenAI as a "search engine" to address questions in medical school: students do not know if they are missing out on critical pieces of information. This is especially noteworthy since different wordings of questions yield different answers. These answers may provide the necessary information, allude to the information requiring follow-up, or not allude to the information. The third category is dangerous since the student will not even know that he or she is missing out on the information. Another key issue is that GenAI does not adequately generate information on complex information, such as the genetic component of multiple sclerosis. These complex areas of science where a definitive answer is not yet known can result in AI delivering false information or result in AI hallucinations. Strong conducted an important experiment to assess ChatGPT's clinical reasoning abilities using clinical cases of varying difficulty levels [21]. He noted that its responses were comparable to those of first- or second-year medical students, but it missed critical details in complex cases involving multisystem conditions.

There is some evidence that ChatGPT can clinically reason similarly to medical students and in-training residents on standardized examinations. Kung et al. evaluated the performance of ChatGPT on USMLE Step 1, Step 2, and Step 3 exams [22]. Through their analysis, the authors found that the GenAI could perform around the passing level for all three exams. As such, the authors concluded that there is potential for GenAI to teach medical students and assist them in passing these exams. Furthermore, the authors concluded that given the GenAI's performance on these exams, there is also potential for it to be used in a clinical setting. In a separate study, Li et al. utilized ChatGPT in virtual objective structured clinical examinations within the field of obstetrics and gynecology [23]. They observed that ChatGPT consistently generated factually accurate and contextually relevant responses to complex and dynamically evolving clinical inquiries. ChatGPT surpassed human candidates in various knowledge domains. In another study, Lum found that ChatGPT achieved scores in the 40th percentile when evaluated against first-year orthopedic surgery residents, in the eighth percentile for second-year residents, and the first percentile for third--, fourth--, and fifth-year residents [24]. These findings suggest that ChatGPT's testing performance is rather comparable to that of a first-year orthopedic surgery resident. The variation in results observed between these studies may be attributed to the specific fields under investigation, highlighting the necessity for further research in various domains within this context.

Generating test questions

In assessing the ability of GenAI to create test questions appropriate for medical students, it was found that ChatGPT had the ability to create high-quality multiple-choice exam questions. Many questions generated were brief clinical scenarios that required answerers to know not just one fact but several facts. This is similar to the questions that students encounter in national licensing exams and various course and clerkship shelf exams. As such, ChatGPT is a useful tool for faculty to create questions or for students to generate questions to test their knowledge. Although ChatGPT can generate high-quality, board-style questions, it also tends to create simpler questions relating to whether or not the answerer knows one specific fact. Overall, since test questions are central to the assessment of medical students throughout their preclinical and clinical curriculum, GenAI can potentially improve question generation and design. This is especially true for schools that rely entirely on faculty-generated questions or a hybrid of NBME and faculty questions. Others have employed ChatGPT to generate clinical case questions, noting its ability to produce plausible and well-structured questions within seconds; however, occasional inaccuracies in the content underscore the importance of subject matter expert review and verification [25].

Applicating to case-based learning

The last aspect of this study involved assessing the potential impact of GenAI in assisting students in case-based learning. Over the last decade, more and more medical schools have adopted a case-based learning model that relies on faculty guiding students through clinical scenarios, a set of questions to be addressed, and defined learning objectives [26]. Given this model, it is conceivable that GenAI has the potential to serve as a personalized guide to students through these scenarios where it would adapt to a student's knowledge level. In assessing the role of GenAI in a problem-based learning scenario, it was noted that responses to questions and prompts included a significant amount of the key learning items outlined in the facilitator guide. For instance, when prompted to consider microorganisms that may cause pneumonia in a 12-year-old, GenAI correctly identified that mycoplasma pneumonia should be considered. Overall, this serves as a positive indicator that GenAI possesses the capability to correctly answer questions in a problem-based learning scenario. As a result, it is possible that it will soon have the ability to guide students through scenarios and identify knowledge gaps for students. However, much onus will be placed on the faculty and administration to properly integrate GenAI in case-based learning.

In his study, Eysenbach highlights the potential for GenAI to create realistic patient scenarios and individualized learning experiences and to summarize studies for quickness and ease of understanding [27]. He explains how a conversation with someone with undiagnosed diabetes may proceed. From there, he continues to prompt the GenAI to provide additional information regarding a physician's conversations with a patient surrounding diabetes, including pathophysiology and management. He notes that ChatGPT is still limited and requires specific prompting that a medical student may not have the appropriate knowledge to ask. In another study, Buhr et al. evaluate ChatGPT's answers to otorhinolaryngology case-based questions compared to consultants' answers [28]. They noted that while ChatGPT provided longer answers, medical adequacy, and conciseness were significantly lower than consultants' answers. These limitations underscore the importance of recognizing that implementing ChatGPT in case-based learning depends on understanding its constraints and developing strategies to address them.

Limitations and future directions

This study assessed ChatGPT's performance in medical education from the authors' perspective. Future research should encompass medical students' perspectives and experiences while examining the short-term and long-term educational impacts of integrating GenAI into the curriculum through comparative study designs. As publicly available GenAI models become more prevalent, comparing ChatGPT against other GenAI models is imperative. Our study's difficulty level of questions and the clinical case were tailored to suit medical students. Caution should be exercised when extrapolating these findings to more complex teaching materials and cases with higher difficulty levels, such as those encountered by residents during training or practicing physicians.

## Conclusions

Overall, GenAI has the potential to impact the medical school curriculum. While not yet comprehensive enough to serve as a repository of information, like a textbook, GenAI has the ability to answer questions, generate test questions, and appropriately respond to prompts in case-based learning scenarios. It performed well in addressing prompts related to epidemiology, diagnosis, and treatment but struggled to generate information on complex topics. It may fail to provide some key information, raising concerns about its suitability as a "search engine" for medical queries. This study offers insights into the current strengths of GenAI and its disadvantages. It can provide guidance to both students and faculty on how best to integrate GenAI, such as ChatGPT, into the current medical school paradigm.

## Appendices

ChatGPT responses to case-based learning prompts and questions.

Session #1.1

You are a Family Practice physician practicing in a small group. You have been taking care of the Coughlin family for several years and know the parents and the four children well. Today the mother brings in two-year old Lotta for a problem visit. It is Monday morning, and Mrs. Coughlin relates that Lotta developed cough and fever over the weekend.

Question #1

What are some possible explanations for her symptoms?

Answer #1

There are several possible explanations for Lotta's cough and fever. Some of the common causes include: Respiratory infections: Lotta may have developed a viral or bacterial respiratory infection, such as the common cold, flu, or pneumonia, which can cause cough and fever. Asthma: If Lotta has a history of asthma or allergies, she may be experiencing an asthma flare-up triggered by a respiratory infection or environmental irritants. Bronchiolitis: This is a viral infection that primarily affects young children and can cause cough, wheezing, and fever. Allergies: If Lotta has a history of allergies, she may be experiencing an allergic reaction to a new allergen, such as pollen or pet dander. Croup: This is a viral infection that primarily affects young children and can cause a bark-like cough, fever, and difficulty breathing. Pertussis: Also known as whooping cough, this bacterial infection can cause a persistent cough, fever, and difficulty breathing. Respiratory syncytial virus (RSV): This is a common viral infection that can cause respiratory symptoms, such as cough and fever, especially in young children. It is important to obtain a thorough medical history, conduct a physical examination, and potentially perform diagnostic tests to determine the cause of Lotta's symptoms and develop an appropriate treatment plan.

Question #2

What questions do you want to ask Mrs. Coughlin?

Answer #2

As a healthcare provider, when presented with a patient with symptoms such as cough and fever, it is important to gather a thorough history from the patient and/or their caregiver. Some questions that could be asked to Mrs. Coughlin include: When did Lotta's symptoms start? Has Lotta had a fever, and if so, how high has it been and for how long? Has Lotta had any other symptoms, such as runny nose, congestion, or difficulty breathing? Has Lotta been exposed to anyone with similar symptoms recently? Has Lotta had any recent vaccinations? Does Lotta have any medical conditions or take any medications? Has Lotta had any recent travel or been in contact with anyone who has recently traveled? Has Lotta been eating and drinking normally? Has Lotta been getting enough rest and sleep? Has there been any change in Lotta's behavior or overall health?

Session #1.2

Lotta started coughing on Saturday morning. The cough is non-productive. It is constant and bothersome throughout the day and has kept her from sleeping. She has also had a poor appetite but is taking juices and milk well. Her temperature has been as high as 101.8°F. Mom has not given any over the counter medications other than Tylenol, which reduces her fever. Lotta has not had any nasal congestion or runny nose, no vomiting or diarrhea. Lotta does attend day care and several of her playmates were out last week with respiratory problems, but no other household members have had cough or cold symptoms.

Lotta has had coughs and colds in the past, Mrs. Coughlin says, “about the same as my other kids,” but this one seems a bit more worrisome to her. She was born full term, has had all of her routine vaccinations, and has no history of surgeries or hospitalizations. She has no allergies to any medications. There is no significant family history.

Question #3

What will you look for on physical examination?

Answer #3

Based on Lotta's symptoms, it is important to perform a thorough physical examination to assess her respiratory status. Some key things to look for during the examination include: General appearance: assess Lotta's overall appearance, including her level of alertness, color, and hydration status. Vital signs: measure Lotta's temperature, heart rate, respiratory rate, and blood pressure. Respiratory examination: listen to Lotta's lungs with a stethoscope for any abnormal sounds such as crackles, wheezes, or diminished breath sounds. Assess for signs of respiratory distress such as increased work of breathing or use of accessory muscles. Head and neck examination: assess Lotta's throat for any signs of redness, swelling, or exudate. Ear examination: assess Lotta's ears for any signs of infection. Abdominal examination: assess Lotta's abdomen for any signs of discomfort, distension, or tenderness. Skin examination: assess Lotta's skin for any rashes, lesions, or signs of dehydration. Based on the results of the physical examination, further diagnostic testing or referral to a specialist may be necessary.

Question #4

How will your approach to examining a two-year-old differ from examining an adult patient?

Answer #4

When examining a two-year-old patient, the approach should be tailored to their developmental stage, age, and temperament. The following differences in approach can be considered: Communication: Communication with a two-year-old is limited, and the examiner may need to rely on observation to obtain information. A calm and friendly tone of voice may help to reassure the child. Parental presence: The presence of a parent or caregiver is often necessary to keep the child calm and cooperative. Observation: Observation of the child's behavior, posture, and movements can provide important diagnostic information. Play-based exam: Examining a child may involve using toys, bubbles, or other objects to help distract them and make the experience more enjoyable. Brief exam: The exam should be kept brief and focused, as the child may become restless or uncooperative. Flexibility: The examiner should be prepared to modify the approach based on the child's temperament, level of cooperation, and developmental stage. Safety: The examiner should ensure the child's safety during the exam, by keeping sharp or hazardous objects out of reach and making sure that the child is properly secured if lying on an exam table.

Session #1.3

Physical Exam:

HR 110, RR 28, BP 100/50, T 100.7°F

Pulse oximeter on room air: 94%

Gen: WDWN toddler, sitting quietly in mother’s lap

HEENT: mucous membranes pink and moist, tympanic membranes pearly grey, no conjunctival erythema, no nasal discharge, pharynx without erythema, no tonsillar enlargement or exudates, no cervical lymphadenopathy

Lungs: 1+ intercostal retractions (link to definition: inward movement of muscles between the ribs; a sign of difficulty with breathing) at rest; inspiratory rales at left base; breath sounds otherwise normal with good air exchange, no wheezes

Cardiac: RRR, no murmurs, rubs, or gallop

Abdomen soft, non-tender, no hepatosplenomegaly, bowel sounds present.

Extremities: warm, well-perfused, 2+ pulses, brisk capillary refill (link: an indicator of tissue perfusion; usually <2 seconds)

Neurologic: Alert, appropriately responsive to exam (bats away stethoscope, clings to mom, cries), moves all extremities

Question #5

What further information would you like?

Answer #5

Based on the physical exam findings, the patient seems to be experiencing respiratory distress. To further assess the patient's condition, it would be helpful to gather additional information, such as: Any recent travel or exposure to individuals with respiratory illnesses Any history of asthma or allergies Any history of recent hospitalizations or medical procedures The duration of the current illness and the progression of symptoms over time Any additional symptoms such as chest pain, abdominal pain, or changes in urinary or bowel habits The patient's immunization history Any medications that the patient is currently taking The patient's recent weight and growth trajectory

Question #6

What tests would you like to order?

Answer #6

Based on the information obtained from the history and physical examination, the following tests can be considered: Pulse oximetry: This can be repeated after the patient is placed on supplemental oxygen to see if the oxygen saturation improves. Chest X-ray: This can help to evaluate the lungs and detect any signs of infection, such as pneumonia. Complete blood count (CBC) with differential: This can help to evaluate for infection or inflammation. Respiratory viral panel: This can help to identify the specific virus causing the respiratory symptoms. Throat culture: This can help to identify any bacterial infections, such as strep throat. The need for any tests would depend on the suspected diagnosis and the severity of the patient's symptoms. Further diagnostic testing may also be necessary depending on the results of initial tests and the progression of the patient's symptoms.

Session #1.4

Image of CXR showing LLL infiltrate.

Question #7

What do you think is wrong with Lotta?

Answer #7

Based on the symptoms and the finding of a left lower lobe infiltrate on the chest x-ray, it is likely that Lotta has a bacterial pneumonia. Pneumonia is an infection of the lungs caused by bacteria, viruses, or other microorganisms. The symptoms of pneumonia include cough, fever, shortness of breath, chest pain, and fatigue. The infiltrate seen on the chest x-ray indicates that there is consolidation of lung tissue, which is a characteristic feature of pneumonia. In Lotta's case, her symptoms of cough, fever, and respiratory distress, along with the presence of an infiltrate on the chest x-ray, suggest a bacterial pneumonia, which is a common cause of community-acquired pneumonia in children. Further tests, such as blood cultures and a complete blood count (CBC), may be needed to confirm the diagnosis and determine the type of bacteria causing the infection.

Question #8

What organisms do you need to cover when treating pneumonia in this age group?

Answer #8

In children aged 2 years and younger with pneumonia, the most common causative organisms are viruses, such as respiratory syncytial virus (RSV) and influenza virus, and bacteria, such as Streptococcus pneumoniae, Haemophilus influenzae type b, and Mycoplasma pneumoniae. Atypical bacteria, such as Chlamydia trachomatis and Chlamydophila pneumoniae, should also be considered in this age group. Therefore, treatment regimens for pneumonia in this age group typically cover both viral and bacterial pathogens.

Question #9

How would this differ if Lotta were a newborn? Or 12-years-old?

Answer #9

The approach to the diagnosis and treatment of pneumonia would be different in a newborn and a 12-year-old compared to a 2-year-old. Newborns are at higher risk of bacterial pneumonia, including Group B Streptococcus, Escherichia coli, Listeria monocytogenes, and viruses such as respiratory syncytial virus (RSV) and herpes simplex virus. Therefore, when evaluating a newborn with suspected pneumonia, broad-spectrum antibiotics effective against these organisms may be initiated immediately. Diagnostic tests such as blood cultures, chest X-ray, and lumbar puncture may also be performed. In contrast, in a 12-year-old child, the common causes of pneumonia include atypical organisms such as Mycoplasma pneumoniae, Chlamydia pneumoniae, and viruses such as influenza. Bacterial causes such as Streptococcus pneumoniae and Haemophilus influenzae are less common. Therefore, the evaluation and management of pneumonia in a 12-year-old would focus on obtaining a detailed history and physical examination to identify the underlying cause of the pneumonia, and targeted antibiotics based on the likely causative organism may be prescribed. Diagnostic tests such as chest X-ray and laboratory tests such as complete blood count (CBC) and inflammatory markers may also be ordered. Overall, the management of pneumonia in each age group would depend on the specific clinical presentation and underlying causes of the disease.

Session #1.5

Lotta is diagnosed with pneumonia and started on Augmentin. You instruct Mom to return to the office in three days if Lotta isn’t doing any better, or sooner if she feels Lotta is getting worse.

Question #10

Why was drug Augmentin chosen? What is its mechanism of action?

Answer #10

Augmentin is a combination antibiotic medication that contains amoxicillin and clavulanate potassium. It is often used to treat bacterial infections, including pneumonia. The choice of Augmentin in Lotta's case may have been based on the likelihood that her pneumonia is caused by bacteria, rather than a virus. The addition of clavulanate potassium to amoxicillin helps to extend its spectrum of activity against certain bacteria that are resistant to amoxicillin alone. Amoxicillin works by inhibiting the formation of the bacterial cell wall, which ultimately leads to bacterial death. Clavulanate potassium works by inhibiting bacterial beta-lactamases, enzymes that can inactivate amoxicillin. It is important to note that the choice of antibiotic and the duration of treatment may vary depending on the specific organism causing the infection, the severity of the illness, and other factors. It is also important to follow the instructions of the prescribing healthcare provider and complete the full course of antibiotics to ensure that the infection is fully treated and to prevent the development of antibiotic resistance.

Session #1.6

Lotta’s mother brings her back two days later (Wednesday), concerned that Lotta’s cough is worsening and her sputum this morning appeared to be blood-tinged. Her fevers are higher, now up to 103.5°F at home. She says Lotta seems to be working harder to breathe, her energy level has decreased. She is not even interested in juices anymore. She has not had a wet diaper since the early morning, which is concerning.

VS: HR 148, RR 55, BP 95/45, T 103.8, O2 sat 88% RA

On physical exam, Lotta appears listless and is in moderate distress. She doesn’t fight your exam as she did on the previous day. On pulmonary exam, her breath sounds are now diminished at the left base, with rales in the left and right lower lobes. Her work of breathing is significantly increased, with 2+ intercostals retractions. Her extremities are cool, pulses are 2+, capillary refill is 4-6 seconds.    

When asked if anyone else at home is ill, Mom again denies any respiratory symptoms in family members. She does state, however, that her teenage son has recently been afflicted with sore red bumps on his skin. He is on the school soccer team and reported to his mother that the bumps were “going around” among the team members. He first developed the skin bumps three weeks ago. The bumps vary in size and location, are red, warm, and tender to the touch Last week two of these lesions were drained in the Emergency Department, and her son was given some antibiotics.

Picture of skin lesion.

Question #11

What studies would you like to order now?

Answer #11

Given the worsening symptoms and the presence of blood-tinged sputum, it is important to evaluate Lotta for any potential complications of pneumonia, such as a pleural effusion or empyema. A chest x-ray should be repeated to assess for any changes in the pneumonia or any new findings. Given the possibility of a bacterial superinfection or co-infection with a different organism, blood cultures and a respiratory viral panel should be obtained. In addition, a complete blood count (CBC) with differential and a basic metabolic panel (BMP) should be ordered to assess for any electrolyte imbalances or signs of dehydration. Finally, given the presence of skin lesions on the family member, a thorough skin examination should be performed on Lotta to assess for any similar lesions, and a culture of any suspicious lesions should be obtained to rule out a possible staphylococcal infection.

Question #12

Is the history of skin lesions in family members significant?

Answer #12

Yes, the history of skin lesions in Lotta's teenage brother is significant because it suggests that there may be a possibility of a skin infection that could be related to Lotta's worsening respiratory symptoms. Some skin infections, such as methicillin-resistant Staphylococcus aureus (MRSA), can cause pneumonia in certain situations, and this possibility should be investigated further.

Question #13

What is the difference between cellulitis and a skin abscess?

Answer #13

Cellulitis is an infection of the skin and subcutaneous tissue that typically presents with erythema, edema, warmth, and tenderness. It is usually caused by bacteria, most commonly Streptococcus pyogenes and Staphylococcus aureus, and can occur anywhere on the body. In contrast, a skin abscess is a localized collection of pus within the dermis or subcutaneous tissue, typically caused by the same bacterial pathogens. Abscesses are usually tender, fluctuant nodules that may be red and warm. The key difference between cellulitis and a skin abscess is the presence of a localized collection of pus within the latter. While cellulitis involves diffuse skin and subcutaneous tissue infection, an abscess represents a more focal infection. Additionally, treatment of these two conditions differs: cellulitis is typically treated with antibiotics, while an abscess may require drainage in addition to antibiotic therapy.

Question #14

Which pathogens are typically responsible for each of these disorders?

Answer #14

Cellulitis and skin abscess can be caused by a variety of pathogens, including bacteria such as Staphylococcus aureus, Streptococcus pyogenes, and Gram-negative bacteria such as Escherichia coli or Klebsiella pneumoniae. However, Staphylococcus aureus is the most common cause of both cellulitis and skin abscess. In cellulitis, the infection is typically caused by bacteria that invade the skin through a break or cut in the skin. Streptococcus pyogenes and Staphylococcus aureus are the most common causes of cellulitis. In some cases, cellulitis may also be caused by other bacteria such as Haemophilus influenzae, Pseudomonas aeruginosa, or group B Streptococcus. In a skin abscess, the infection is often caused by Staphylococcus aureus, especially methicillin-resistant Staphylococcus aureus (MRSA). Other bacteria such as Streptococcus pyogenes, Pseudomonas aeruginosa, and Gram-negative bacteria can also cause skin abscesses.

Session #1.7

Lab results: CBC, BMP, coagulation profile; blood culture pending.

**Table 3:** Lab results

Test	Patient	Normal
Complete Blood Count with Differential		
White blood cells	18,000	4,800-10,800/µL
Neutrophils	66%	36-66%
Bands	14%	0
Lymphocytes	17%	24-44%
Monocytes	3%	1.7-9.3%
Hemoglobin	12 g/dL	12-16 g/dL
Hematocrit	37%	37-47%
Platelets	180,000	140,000-440,000/µL
Basic Metabolic Panel		
Sodium	141	133-142 mmol/L
Potassium	4.2	4.0-6.5 mmol/L
Chloride	109	95-110 mmol/L
Bicarbonate	15	22-30 mmol/L
Blood urea nitrogen	25	8-28 mg/dL
Glucose	108	60-110 mg/dL
Creatinine	0.8	0.3-0.7 mg/dL
Coagulation Profile		
Prothrombin time	12.4	8.9-21.1 sec
Partial thromboplastin time	28.1	<30 sec
Fibrinogen	285	180-360 mg/dL

CXR 2: increased density of left basilar opacification with small pleural effusion; new RLL opacification.

Lotta is admitted to the hospital for IV antibiotics. She is given two 20 cc/kg NS boluses and placed on intravenous fluids for hydration. Ceftriaxone and oxacillin are started.

Question #15

What is the spectrum of coverage and mechanism of action of each of the antibiotics chosen?

Answer #15

Ceftriaxone is a third-generation cephalosporin antibiotic with a broad spectrum of coverage against gram-positive and gram-negative bacteria. It works by inhibiting bacterial cell wall synthesis. Specifically, it binds to penicillin-binding proteins (PBPs) and inhibits the final transpeptidation step of peptidoglycan synthesis in bacterial cell walls, leading to cell lysis and death. Oxacillin is a beta-lactam antibiotic in the penicillin class, with a narrow spectrum of coverage against gram-positive bacteria, specifically against penicillinase-producing Staphylococcus aureus. It works by binding to PBPs, leading to inhibition of peptidoglycan synthesis and cell wall formation. Together, ceftriaxone and oxacillin provide broad-spectrum coverage against both gram-positive and gram-negative bacteria, including potential pathogens such as Streptococcus pneumoniae, Haemophilus influenzae, and methicillin-sensitive Staphylococcus aureus.

Question #16

Do you agree with the choice of IV antibiotics? Are there any potential pathogens not being covered?

Answer #16

Based on the information provided, the choice of IV antibiotics (ceftriaxone and oxacillin) seems appropriate for the initial empiric treatment of Lotta's suspected community-acquired pneumonia and possible sepsis. Ceftriaxone has a broad spectrum of coverage, including common respiratory pathogens such as Streptococcus pneumoniae, Haemophilus influenzae, and Moraxella catarrhalis. Oxacillin is a penicillinase-resistant penicillin that covers Staphylococcus aureus, including methicillin-sensitive strains. However, there are some potential pathogens that may not be covered by these antibiotics. For example, if Lotta's blood culture comes back positive for gram-negative bacteria, the coverage of ceftriaxone may need to be adjusted based on the susceptibility results. Additionally, if Lotta's skin lesion is found to be a soft tissue abscess, the coverage for MRSA may not be adequate with oxacillin alone, and an additional agent such as vancomycin or daptomycin may be needed.

Question #17

What else might be done to treat Lotta’s illness? Why do you think Lotta is not responding to therapy?

Answer #17

Based on the information provided, Lotta's illness appears to be complicated and potentially life-threatening. In addition to the IV antibiotics, she may require additional interventions such as drainage of any abscesses or infected fluid collections, as well as supportive care such as oxygen supplementation and mechanical ventilation if her respiratory status deteriorates. If Lotta is not responding to the chosen antibiotics, it is possible that she is infected with a resistant strain of bacteria or a different type of organism altogether. In this case, her treatment may need to be adjusted based on the results of her blood culture and other diagnostic tests. It is also possible that there are other underlying factors contributing to her illness, such as immunosuppression or other medical conditions, which may need to be addressed in order to improve her response to therapy.

Session #1.8

Over the next 24 hours, Lotta’s condition continues to deteriorate. She is tachypneic and is visibly working harder to breathe. Her oxygen saturations are lower despite more aggressive oxygen therapy. She is now on 6 Liter O2 by face mask with O2 saturations of 87%. She is quite listless and barely cries when a new IV is placed. Her fever is persistently >102° F, HR persistently in the 160s, RR in the 50s and 60s. Her morning labs show that her WBC has risen to 34,000 with 56% segmented neutrophils, 28% bands, 16% lymphocytes. Her CXR is worsening:

Exhibit 4: Dense left lower lobe opacification with suggestion of increase in size of pleural effusion. Decubitus film with little change suggests loculation of fluid (not free flowing).

During a particularly violent coughing spell, a sputum sample had been obtained and sent for gram stain and culture.

Exhibit 5: Gram positive cocci in pairs and clusters.

The lab reports that her sputum is growing coagulase positive Staphylococcus, with further identification and sensitivities to follow.

Question #18

How reliable is a sputum sample for identification of a respiratory pathogen? Is there a “gold standard” for obtaining a respiratory sample for gram stain and culture?

Answer #18

Sputum samples can be reliable for identification of respiratory pathogens if they are properly collected and processed. The gold standard for obtaining a respiratory sample for gram stain and culture is the collection of a bronchoalveolar lavage (BAL) or protected specimen brush (PSB) sample, which is obtained by bronchoscopy. These methods have a higher diagnostic yield and are less likely to be contaminated by upper respiratory tract flora than sputum samples. However, they are more invasive and require specialized equipment and expertise, which may not be readily available in all clinical settings. Therefore, sputum samples are often used as an initial diagnostic tool for respiratory infections, but their results should be interpreted with caution and in the context of the patient's clinical presentation and other laboratory findings.

Question #19

What are possible reasons for Lotta’s failure to respond to therapy?

Answer #19

There could be several possible reasons why Lotta is failing to respond to therapy, including: Inadequate antibiotic coverage: Despite receiving two broad-spectrum antibiotics, it is possible that the pathogen causing Lotta's infection is resistant to one or both of these antibiotics, or that the antibiotic dosing is inadequate for her condition. Delayed treatment: If Lotta's infection had been present for some time before treatment was initiated, the infection may have progressed to a severe stage that is more difficult to treat. Underlying health conditions: Lotta's pre-existing health conditions, such as her Down syndrome and cardiac anomalies, could make her more vulnerable to severe infections that are harder to treat. Immune compromise: Individuals with certain medical conditions, such as immunodeficiencies or autoimmune disorders, may have a weakened immune system that is less able to fight off infections. Infection caused by a particularly virulent pathogen: Some pathogens are more aggressive and can cause more severe infections that are harder to treat. Complications from the infection: Infections can cause complications such as sepsis, which can lead to multi-organ failure and other serious health issues that may be difficult to manage. It is important to conduct further investigations to determine the cause of Lotta's deteriorating condition and adjust treatment as necessary.

Question #20

What further imaging or other diagnostic studies would be helpful at this point?

Answer #20

Given Lotta's clinical deterioration, worsening CXR findings, and persistent fever, additional imaging and diagnostic studies may be necessary to further evaluate and manage her condition. One option is to obtain a CT scan of the chest, which can provide more detailed information on the extent and location of the pulmonary infiltrates and effusions. This may help guide further treatment and potential interventions, such as drainage of the pleural effusion or lung biopsy. Another option is to perform a bronchoscopy with bronchoalveolar lavage (BAL), which can obtain a more specific and accurate sample of the respiratory tract for culture and sensitivity testing. This can help identify the specific pathogen responsible for Lotta's infection and determine the most appropriate antibiotic treatment. Additionally, BAL can help diagnose other potential causes of Lotta's respiratory symptoms, such as aspiration or airway obstruction. Other possible diagnostic studies may include a repeat blood culture to monitor for persistent bacteremia, serologic testing for viral and atypical bacterial pathogens, and assessment of Lotta's cardiac function and hemodynamics. The choice of additional diagnostic tests will depend on the clinical suspicion and the course of Lotta's illness.

Question #21

How will you revise your treatment plan now?

Answer #21

Given the worsening of Lotta's condition and the identification of coagulase-positive Staphylococcus in her sputum culture, it is likely that she has developed a bacterial pneumonia caused by this organism. The sensitivity results from the culture can guide the choice of antibiotics. It is important to note that some strains of Staphylococcus can be resistant to many antibiotics, including methicillin-resistant Staphylococcus aureus (MRSA), which requires different treatment. In addition, given the persistence of Lotta's fever and worsening CXR findings, it may be helpful to obtain a CT scan of her chest to further evaluate the extent and severity of her pneumonia and any potential complications such as an abscess or empyema. Lotta may also require more aggressive respiratory support such as noninvasive ventilation or intubation and mechanical ventilation. Close monitoring of her vital signs, oxygenation, and fluid status is necessary. Therefore, I would recommend revising Lotta's treatment plan by starting her on antibiotics that are effective against coagulase-positive Staphylococcus based on the sensitivity results. It may be necessary to switch from the current antibiotics to a different regimen that covers this organism. I would also recommend obtaining a CT scan of her chest and considering more aggressive respiratory support. Close monitoring of her vital signs and fluid status should continue, and further supportive measures such as intravenous fluids, electrolyte replacement, and nutritional support may also be necessary.

Session #1.9

Concerned that Lotta is progressing towards respiratory failure, you arrange for transfer to the local children’s hospital. You review Lotta’s course with the accepting physician. After hearing her story, the physician states he is concerned about possible methicillin-resistant Staph aureus (MRSA) and suggests starting Lotta on a new antibiotic while the transport team is being mobilized.

Question #22

What is the mechanism of resistance in MRSA?

Answer #22

Methicillin-resistant Staphylococcus aureus (MRSA) is a strain of bacteria that is resistant to many beta-lactam antibiotics, including methicillin, penicillin, and cephalosporins, due to the acquisition of the mecA gene. This gene encodes for a unique penicillin-binding protein called PBP2a or PBP2' that has a low affinity for beta-lactam antibiotics, making them ineffective in treating MRSA infections. The mecA gene is located on a mobile genetic element called staphylococcal cassette chromosome mec (SCCmec), which can be transferred between different strains of Staphylococcus aureus, facilitating the spread of resistance. MRSA infections can be challenging to treat and often require alternative antibiotics, such as vancomycin, daptomycin, or linezolid.

Question #23

What is the epidemiology of MRSA, and how has it changed over the past two decades?

Answer #23

Methicillin-resistant Staphylococcus aureus (MRSA) is a type of bacteria that is resistant to many antibiotics, including methicillin, which is a type of penicillin. MRSA is a significant public health concern because it is associated with increased morbidity, mortality, and healthcare costs. The epidemiology of MRSA has changed significantly over the past two decades. In the past, MRSA infections were mainly associated with healthcare settings, such as hospitals and nursing homes. These healthcare-associated MRSA (HA-MRSA) infections tended to occur in patients who were older, sicker, or had weakened immune systems. However, in recent years, a new strain of MRSA has emerged that is associated with community settings, such as schools, sports teams, and correctional facilities. These community-associated MRSA (CA-MRSA) infections tend to occur in healthy individuals who have not been hospitalized or undergone invasive medical procedures. CA-MRSA strains are also more virulent and capable of causing severe infections, such as necrotizing pneumonia, skin and soft tissue infections, and sepsis. The emergence of CA-MRSA has led to increased concern about the spread of MRSA in the community and the need for enhanced infection control measures. Healthcare facilities have implemented measures to prevent the spread of MRSA, such as hand hygiene, screening of high-risk patients, and isolation precautions. In the community, measures such as good hygiene, prompt treatment of infections, and avoiding close contact with infected individuals can help prevent the spread of MRSA.

Question #24

How is “hospital associated” MRSA different from “community associated” MRSA? How does that affect antibiotic therapy?

Answer #24

Hospital-associated MRSA (HA-MRSA) and community-associated MRSA (CA-MRSA) are different strains of MRSA with different epidemiological characteristics and antibiotic susceptibility patterns. HA-MRSA strains are typically found in healthcare settings, such as hospitals and long-term care facilities. These strains have a higher degree of antibiotic resistance and are more likely to cause severe infections in patients who are already ill or have weakened immune systems. HA-MRSA strains are often resistant to multiple antibiotics, including beta-lactams, macrolides, and aminoglycosides. Therefore, treatment of HA-MRSA infections may require the use of alternative antibiotics, such as vancomycin, linezolid, or daptomycin. In contrast, CA-MRSA strains are typically found in the community, outside of healthcare settings. These strains are often more susceptible to antibiotics and are associated with skin and soft tissue infections in healthy individuals. CA-MRSA strains are often susceptible to antibiotics such as trimethoprim-sulfamethoxazole (TMP-SMX) and tetracyclines and may not require treatment with stronger antibiotics like vancomycin. It is important to distinguish between HA-MRSA and CA-MRSA because the choice of antibiotic therapy may differ based on the strain of MRSA. If there is a high suspicion for HA-MRSA, initial empiric therapy may need to be broader and cover for resistant organisms, while CA-MRSA may be treated with more targeted and less broad-spectrum antibiotics.

Question #25

What antibiotic would you recommend for Lotta?

Answer #25

The choice of antibiotic for Lotta would depend on the susceptibility of the MRSA strain identified by culture and sensitivity testing. In general, vancomycin is often considered the drug of choice for the treatment of MRSA infections, including pneumonia. Other options may include linezolid, daptomycin, or ceftaroline, depending on local resistance patterns and the severity of the infection. It is important to obtain the sensitivity report before selecting an antibiotic to ensure the most appropriate therapy.

Session #2.1

Lotta is transferred to the children’s hospital and admitted to the Intermediate Care Unit (the “step-down” to the Intensive Care Unit).

On arrival, Lotta is placed on 100% O2 via BiPAP, a tight-fitting face mask that allows delivery of both inspiratory and expiratory (“bi-level”) positive pressure). Her O2 saturation improves to 94%. She remains tachypneic with moderately labored respirations. Her care team places her on vancomycin and ceftriaxone and obtains a computerized tomography (CT) scan of the chest. Lotta is placed on “resistant organism precautions” (link: specific guidelines exist to prevent the spread of resistant organisms to other patients or hospital personnel. These include wearing gowns and gloves whenever entering the room of a patient with, or suspected of having, MRSA)) for presumed MRSA infection.

Question #26

What are potential toxicities of vancomycin? How are these avoided?

Answer #26

Vancomycin can cause several toxicities, including kidney damage, red man syndrome, and ototoxicity (damage to the inner ear leading to hearing loss and balance problems). To avoid these toxicities, vancomycin levels should be monitored closely and adjusted based on kidney function. Infusion should be given slowly, over at least an hour, to decrease the risk of red man syndrome. Additionally, patients should be monitored for symptoms of ototoxicity, and the drug should be discontinued if symptoms occur.

Question #27

Why is Lotta in isolation? How long will she need to stay in isolation?

Answer #27

Lotta is in isolation for presumed MRSA infection, as she has a positive sputum culture for coagulase-positive Staphylococcus aureus. MRSA is a type of Staphylococcus aureus that is resistant to many antibiotics, making it difficult to treat. Isolation precautions are necessary to prevent the spread of MRSA to other patients and healthcare workers. The duration of isolation precautions depends on the patient's clinical course and infection control policies at the hospital. Generally, patients with MRSA are placed on contact precautions until they are no longer considered infectious. This may require multiple negative cultures to confirm clearance of the organism. The healthcare team will follow hospital protocols and guidelines to determine when Lotta can be removed from isolation precautions.

Session #2.2

CT of chest: left lung collapse; large left pleural effusion; mediastinal shift to right secondary to effusion; scattered areas of consolidation within right lung.

Pediatric surgery is consulted to assist with management of the pleural effusion. Lotta is taken to the operating room for drainage of the fluid along with placement of a thoracostomy tube (link: a flexible plastic tube inserted into the pleural space through the side of the chest to drain air or fluid).

Question #28

Are there alternative strategies available for management of a pleural effusion? Why was a thoracostomy tube left in place?

Answer #28

There are alternative strategies available for the management of a pleural effusion. Some of these include observation, needle aspiration, and chest tube placement. Observation is typically reserved for small, asymptomatic effusions, while needle aspiration is typically used for small to moderate effusions. However, in cases of larger effusions or those that are causing significant respiratory distress, chest tube placement is the preferred method of drainage. This is because chest tube placement allows for continuous drainage of fluid or air, which can be important in cases of large or loculated effusions. In Lotta's case, a thoracostomy tube was likely left in place because of the size and location of her effusion, as well as the fact that she was experiencing significant respiratory distress. The thoracostomy tube allows for continuous drainage of fluid from the pleural space, which can help to relieve the pressure on the lungs and improve respiratory function. Additionally, the tube allows for ongoing monitoring of the fluid drainage, which can be important in guiding further management of the effusion.

Question #29

What studies should be performed on the pleural fluid?

Answer #29

Several studies can be performed on the pleural fluid to aid in the diagnosis and management of the patient. These studies include: Cell count and differential: This can help identify the presence of infection, inflammation, or malignancy. Gram stain and culture: This can help identify any bacterial or fungal infections. Biochemical analysis: This can include pH, glucose, lactate dehydrogenase (LDH), and protein levels, which can help differentiate between transudative and exudative effusions and identify specific causes of effusions. Cytology: This can help identify the presence of cancer cells. Microbiologic studies: These include PCR for viral or bacterial DNA, and serologic tests for infectious agents such as Mycoplasma pneumoniae or Legionella pneumophila. The specific studies performed may vary depending on the patient's clinical presentation and suspected underlying cause of the effusion.

Question #30

What are some potential causes of a pleural effusion?

Answer #30

There are many potential causes of pleural effusion, including: Congestive heart failure Pneumonia or other lung infections Lung cancer Tuberculosis Autoimmune diseases, such as lupus or rheumatoid arthritis Kidney or liver failure Pulmonary embolism Pancreatitis Trauma or injury to the chest Certain medications The cause of the pleural effusion is often determined by analyzing the pleural fluid and considering the patient's medical history and clinical presentation.

Question #31

What do you think the tests will show in Lotta’s case?

Answer #31

It is difficult to determine the exact results of the tests on Lotta's pleural fluid without additional information. However, based on Lotta's clinical presentation, history, and imaging studies, the pleural fluid is likely to be consistent with an infectious etiology, such as an empyema or complicated parapneumonic effusion. The fluid may also show evidence of inflammation, such as an elevated white blood cell count and levels of inflammatory markers. However, other causes of pleural effusion, such as malignancy or congestive heart failure, cannot be completely ruled out without additional diagnostic testing.

Section #2.3

250 cc of purulent fluid are evacuated from the pleural space at the time of placement. The tube is secured and placed at 20 cm H2O wall suction.

CXR with chest tube.

Initial analysis of the [Pleural] fluid shows:

Gram stain: 3+ WBC (primarily PMNs) with 2+ gram positive cocci in pairs and clusters

**Table 4:** Pleural fluid analysis

Test	Patient	Normal
White blood cells	68,320/mm3	<10,000/mm3
Polymorphonuclear leukocytes	78%	-
Lymphocytes	22%	-
Red blood cells	1,260	-
Lactate dehydrogenase	682 IU/L	<200 IU/L
pH	7.02	
Protein	4.6 gm/dL	<3.0 gm/dL
Glucose	37 mg/dL	>60 mg/dL

Following drainage of the pleural fluid, Lotta’s work of breathing decreases significantly. She is able to come off BiPAP on postoperative day four and is placed on nasal cannula. Her progress from there is slow, but her fever curve gradually improves, and her WBC returns to normal. She gradually regains her appetite.

Sensitivities on her sputum and pleural fluid confirm that she has Methicillin resistant Staph aureus (MRSA). Sensitivities of her organism show that it is sensitive to Clindamycin, D-test negative. She is switched to this antibiotic to complete a 14-day course. Her chest tube drainage diminishes, and the tube is removed on postoperative day 8. She is discharged home after completing her antibiotics and after her oxygen requirement resolves, 2 weeks after her transfer to the tertiary care center.

Question #32

What is the significance of the “D-test”?

Answer #32

The D-test is a test used to determine inducible clindamycin resistance in Staphylococcus aureus. It is performed by placing an erythromycin disc and a clindamycin disc on a culture plate, then looking for a "D" shaped region of flattened growth that forms around the clindamycin disc adjacent to the erythromycin disc. This indicates that the bacteria are resistant to erythromycin and may also be resistant to clindamycin, even if the initial test showed that it was susceptible to clindamycin. This is important to know because giving clindamycin to a patient with inducible resistance can lead to treatment failure and the development of further antibiotic resistance.

Question #33

Will Lotta need to be isolated from other family members when she returns home?

Answer #33

It depends on the hospital's policy and the specific situation of the patient. Since Lotta was diagnosed with MRSA, there is a possibility that she may still be colonized with the bacteria, which means she may still carry the bacteria on her skin or in her nose without having an active infection. In this case, the hospital may recommend precautions to prevent the spread of MRSA to other family members or the community. These precautions may include practicing good hand hygiene, avoiding close contact with others, and using dedicated personal items like towels and bed linens. The hospital may also recommend further testing to determine if Lotta is still colonized with MRSA before she is discharged home. Ultimately, the decision to isolate Lotta or take other precautions would be made by the treating healthcare team in consultation with the patient's family and in accordance with hospital policies and guidelines.

Question #34

Does the brother with a history of skin abscesses need further testing or treatment?

Answer #34

It would be advisable to evaluate Lotta's brother with a history of skin abscesses for MRSA colonization. MRSA can colonize the skin or nasal passages without causing an active infection. Testing for colonization can be done by obtaining swabs from the skin or nose and culturing the samples. If colonization is confirmed, appropriate treatment measures, such as decolonization, may be recommended to reduce the risk of transmission and prevent future infections. It is recommended to consult a healthcare provider for further evaluation and management.
